# How do moral hazard behaviors lead to the waste of medical insurance funds? An empirical study from China

**DOI:** 10.3389/fpubh.2022.988492

**Published:** 2022-10-26

**Authors:** Yinghua Qin, Jingjing Liu, Jiacheng Li, Rizhen Wang, Pengfei Guo, Huan Liu, Zheng Kang, Qunhong Wu

**Affiliations:** ^1^Department of Social Medicine, School of Public Health, Health Management College, Harbin Medical University, Harbin, China; ^2^Department of Health Economy and Social Security, College of Humanities and Management, Guilin Medical University, Guilin, China

**Keywords:** moral hazard, medical insurance fund, social network, qualitative comparative analysis, China

## Abstract

**Objective:**

The huge loss of health insurance funds has been a topic of concern around the world. This study aims to explore the network of moral hazard activities and the attribution mechanisms that lead to the loss of medical insurance funds.

**Methods:**

Data were derived from 314 typical cases of medical insurance moral hazards reported on Chinese government official websites. Social network analysis (SNA) was utilized to visualize the network structure of the moral hazard activities, and crisp-set qualitative comparative analysis (cs/QCA) was conducted to identify conditional configurations leading to funding loss in cases.

**Results:**

In the moral hazard activity network of medical insurance funds, more than 50% of immoral behaviors mainly occur in medical service institutions. Designated private hospitals (degree centrality = 33, closeness centrality = 0.851) and primary medical institutions (degree centrality = 30, closeness centrality = 0.857) are the main offenders that lead to the core problem of medical insurance fraud (degree centrality = 50, eigenvector centrality = 1). Designated public hospitals (degree centrality = 27, closeness centrality = 0.865) are main contributor to another important problem that illegal medical charges (degree centrality = 26, closeness centrality = 0.593). Non-medical insurance items swap medical insurance items (degree centrality = 28), forged medical records (degree centrality = 25), false hospitalization (degree centrality = 24), and overtreatment (degree centrality = 23) are important immoral nodes. According to the results of cs/QCA, low-economic pressure, low informatization, insufficient policy intervention, and organization such as public medical institutions, were the high-risk conditional configuration of opportunism; and high-economic pressure, insufficient policy intervention, and organizations, such as public medical institutions and high violation rates, were the high-risk conditional configuration of risky adventurism (solution coverage = 31.03%, solution consistency = 90%).

**Conclusion:**

There are various types of moral hazard activities in medical insurance, which constitute a complex network of behaviors. Most moral hazard activities happen in medical institutions. Opportunism lack of regulatory technology and risky adventurism with economic pressure are two types causing high loss of funds, and the cases of high loss mainly occur before the government implemented intervention. The government should strengthen the regulatory intervention and improve the level of informatization for monitoring the moral hazard of medical insurance funds, especially in areas with low economic development and high incident rates, and focus on monitoring the behaviors of major medical services providers.

## Introduction

Moral hazard is a relatively broad term in health insurance. In 1963, Arrow first proposed the concept of moral hazard and expressed it as the insured using more health care services to treat specific diseases than the uninsured (1). From the perspective of the demand side, moral hazard was defined as excessive demand for health investment caused by having health insurance (2). Nyman's book, *The Theory of Demand for Health Insurance*, presented that “moral hazard is sometimes represented by expensive, life-saving treatments for the seriously ill and sometimes by discretionary, even frivolous, procedures for the healthy” (3). It indicated that moral hazard leads to behaviors such as medical abuse and rising costs. On the other hand, some scholars think that moral hazard is a problematic ethical practice that increases opportunities for individual profit while transferring the risk of loss to the group, which explained the reasons for the out-of-control healthcare costs (4). According to Jou, opportunist insurance fraud appears to be portrayed as a “moral war” between “rotten” businesses and “dishonest” customers, and moral hazard by the insured is believed to be the cause of all insurance fraud (5). As the insurance industry has grown, the literature is full of normative expressions of moral hazard, which is described as “stealing or lying or magnifying minor harm, or delay in being able to return to work, misrepresentation, and negligence”. It can be seen that the modern concept of moral hazard involves both correlation and causation: insurance changes behavior and induces claims (6). So moral hazard behaviors in medical insurance funds refer to actors using illegal means deliberately and causing undesirable consequences, and it brings a great threat to the safety of the operation, management, and use of medical insurance funds.

Moral hazard behaviors such as fraud, waste, and abuse in the medical insurance market have caused a major financial impact on the development of health care causes worldwide (7). Global Health Care Anti-Fraud Network estimated that $260 billion, ~6% of global health care spending is lost due to fraud each year (8). The United States accounts for 3–10% of its health care expenditures each year caused by fraud claims and medical abuse, costing between $100 and $300 billion (9, 10). The NHS Fraud Authority (NHS CFA) predicted that in the UK the NHS costs around £1.27 billion due to moral hazards such as fraud annually (11). According to the cases of sanctioning healthcare professionals in South Africa, 51.7% of ethical transgressions were for insurance fraud, and the amount lost per year is about ZAR 13 billion in private healthcare sectors (12, 13). Statistics for some Asian countries are also appalling. India's healthcare industry is losing around Rs 600–800 crore annually due to fraudulent claims (14). According to a recent study, nearly 100,000 people were arrested in South Korea for medical insurance fraud, and the amount of fraud was as high as KRW 898,592 million (15). China also faces the same problem of wasting medical insurance funds. In 2018, National Health Security Administration (NHSA) launched a special action against the fraudulent acquisition of medical insurance funds. From 2019 to 2021, NHSA investigated and punished 1.07 million institutions and 104,900 insureds, resulting in a total loss of 57.285 billion yuan in medical insurance funds (16–18). Unfortunately, for formal or informal reasons, there is currently limited research evidence on immoral phenomena in health insurance worldwide (15, 19).

## Methods of identifying and exploring moral hazards in medical insurance

Identification of moral hazard is a problem of categorizing behaviors and perpetrators, to respond to the legality of health care claims. Social network analysis (SNA) as an analysis method, focuses on the structure and closeness of relationships among actors with different activities in an action network and visualizes various relationships in a social network, which could be applied to analyze moral hazard behaviors (20). Scholars such as Šubeljetal, Soheil Jamshidi, and Óskarsdóttir M used SNA to test insurance fraud and proved that the model with network characteristics performs well in detecting fraud (21–24). SNA provides a new research path for identifying moral hazard behaviors of medical insurance.

Although many studies have reported that medical insurance fund losses are caused by moral hazards, we have not found relevant research through analysis of cases to explore the causes of medical insurance fund loss (25, 26). Qualitative comparative analysis (QCA) may be a good try, which is a method developed by Ragin and is case-oriented, systematically compare cases based on the properties of cases and their relationship with specific results (27–29). Compared to traditional regression analysis, the advantage of QCA can analyze small sample cases, and perform combined analysis on the causes or conditions that lead to the results (30). It is fit to deal with the results of multiple conditions and multiple factor combinations in complex social situations, and it can also analyze the conditional combinations that lead to the asymmetric causality of social phenomena (31, 32). In recent years, QCA begun to explore the field of medical and health care research gradually (29, 33–35).

## Research status of moral hazards in China's medical insurance fund

Although the Chinese government has increased its determination to combat the moral hazard of medical insurance in recent years, the research on this issue in the academic field still needs to accelerate the pace. Feng used a small number of medical insurance moral hazard cases to conduct a qualitative analysis, and the defects of BMIS and fund management bugs are the main causes (36–38). Yang and Xiao analyzed the core logic of fraud insurance from the perspective of moral hazard and put forward the anti-fraud system of the medical insurance fund based on the process (39). However, there are few concerns on comprehensive identification and attributional analysis of all possible moral hazard behaviors of the medical insurance fund.

Overall, moral hazard affects the normal operation of medical insurance funds. Identification of moral hazard behaviors and their occurrence mechanism is the first step in risk management (12). Therefore, the study aims to visualize the relationship of stakeholders and their moral hazard actions through social network analysis (SNA), and further explore the combination of conditions leading to funding loss by using crisp-set qualitative comparative analysis (cs/QCA) based on diamond fraud theory, to help Chinese governance to efficiently predict and investigate immoral behaviors in medical insurance and propose specific strategies to reduce moral hazards in the operation of medical insurance funds.

## Materials and methods

### Study design

**Figure 1:**
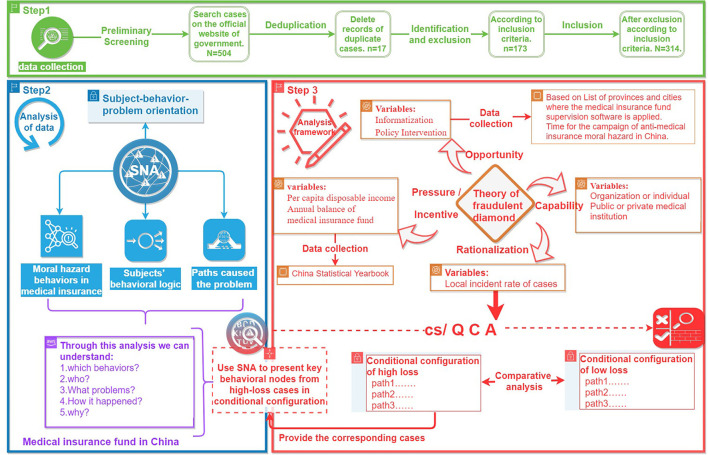
Flowchart of case analysis in study design.

A mixed research method combined SNA and cs/QCA is used to analyze the moral hazard behaviors in medical insurance funds. The study design is based on the subject–behavior–problem orientation to collect the basic data of typical cases, and secondary indexes are collected according to the four dimensions of the theory of fraudulent diamond proposed by Wolfe (40). The behavioral logic of medical insurance moral hazard and the conditional configuration of the causes of losses are identified. First, we use SNA to explore the behavioral network characteristics of moral hazard and describe the path of different subjects' results in moral hazard problems. Second, cs/QCA is used to explore factors associated with different levels of losses of the funds. Finally, combined with the results from cs/QCA and SNA, the important behavioral nodes and paths resulting in high losses are determined (Figure 1).

### Data collection

To identify the networks and drivers of immoral behaviors in medical insurance, this study focuses on medical insurance violation cases published on the official websites of Chinese central government departments from 2017 to 2021. A sample of 314 cases of medical insurance violations was downloaded and collected through the designated disclosure channels of medical insurance funds in China, such as the national Audit office of the people's republic of China and the national healthcare security administration exposure platform (Table 1). Criteria for sample inclusion: (a) involves the moral hazard activities that violated laws or regulations; (b) clear information of the violator; (c) specifically describes the methods that violated the regulations or laws; (d) reports the punishment result of moral hazard behaviors.

**Table 1:** Basic information of typical cases.

**Variable** **category**	**Variable name**	**Frequency**	**%**	**Variable** **category**	**Variable name**	**Frequency**	**%**
Subjects of action	Insured	84	26.75	Types of problems	Insurance fraud	201	64.01
	Designated public hospital	65	20.70		Illegal medical charges	54	17.20
	Designated private hospital	56	17.83		Repeat insurance	28	8.92
	Designated primary medical institution	41	13.06		Use Medicare Fund in Violation of Rules	17	5.41
	Designated pharmacy	34	10.83		Underpayment of premium	16	5.10
	Employer	25	7.96		Irregular management	12	3.82
	Medical insurance agency	15	4.78		Refusal or omission of insurance	8	2.55
	Medical staff	11	3.50		Conduct medical services in violation of regulations	6	1.91
	Non-Insured	7	2.23		Defrauding the qualifications of designated services	1	0.32
	Non designated pharmacy	7	2.23	Action	Behaviors (Supplementary File 1)		
	Hospital administrator	4	1.27	Source of cases	National healthcare security administration (Exposure platform)	174	55.41
	Non-medical institution	3	0.96		National audit office of the people's republic of China	138	43.95
	Medical insurance manager	2	0.64		Ministry of human resources and social security of the people's republic of China	1	0.32
	Other subjects	6	1.91		National health commission of the people's republic of China	1	0.32

^a^Other subjects include School/Drug trafficker/Intermediary agency/Non designated hospital/Finance Department/Hospital scalper.

A total of 314 typical cases that met the conditions were finally included. The key variables of cases were extracted, coded, and standardized. To avoid the subjectivity of information extraction, the key variables extraction was carried out independently by two researchers. Before starting information classification, researchers pre-coded the cases to ensure that they reached a basic consensus on information interpretation, the extraction process as well as coding methods (Table 1).

## Methods

### Social network analysis

This study uses social network analysis (SNA) to visualize and analyze the network structure of the moral hazard actions, the network characteristics of cooperation between stakeholders, as well as the relationship between roles and behaviors. In this section, based on the subject–behavior-problem orientation, we use three elements of information extracted from sample cases: subjects of action, behaviors, and types of problems (Table 1). A visual social network composed of nodes (actors or actions), edges connecting nodes, and weights of edges can show the paths of communication and behavioral interaction between different actors. Finally, the key nodes in the network that affected the governance outcome of unethical behavior were discussed. The Gephi 9.2 software was used to draw the social network graph to visualize various relationships and calculate statistical parameters (41). The main indicators of SNA are as follows: degree centrality, closeness centrality, betweenness centrality, eigenvector centrality, and modularity.

a. Degree centrality (DC) means that counts of links (edges linking adjacent nodes) about the relationship between cooperators or various immoral behaviors are used to measure the centrality of nodes in the network (42).
b. Closeness centrality (CC) means the degree of difficulty from one node to other nodes.
c. Betweenness centrality (BC) refers to the number of times that a node acts as a bridge for the shortest path between two other nodes, that is, the intermediary effect of the node.
d. Eigenvector centrality (EC) refers to the importance of the neighbors of a node, which is used to measure the influence of the node in the network.
e. Modularity refers to the degree to which nodes tend to cluster, and the purpose is to identify the communities formed in the social network (42).

### Qualitative comparative analysis using crisp sets

In this study, we noticed the amount of loss involved in cases. Hence, we further explore the combination of conditions leading to different levels of losses in moral hazard using cs/QCA. The basic idea of QCA is to use set theory and Boolean algebra (and or not) to identify binary data patterns (43). Necessary conditions and sufficient conditions are two important terms, when the necessary conditions and sufficient conditions are identified, the results can be identified (44, 45). Consistency and coverage are two important indicators, with values ranging from 0 to 1. When the consistency of a single condition or a combination of conditions is >0.9, it can be reasonably considered to be a necessary condition leading to the appearance of the result. The coverage rate indicates the interpretation range of the result variable by the combination of conditions. By analyzing the parameters of the consistency and coverage of the condition organization and the results, the key conditions, and paths leading to the results are determined, and consistency is considered meaningful at a threshold of 0.8 (43, 46).

### Setting condition variables in cs/QCA

By reviewing the research on insurance fraud closely related to medical insurance moral hazard, criminologist Cressey proposed a triangle model that leads to fraud, arguing that pressure (or motive), perceivable opportunity, and rationalization of actors are the three elements that lead to fraud (47). Wolfe further proposed the theory of fraudulent diamond, which states that potential violators must also have the corresponding capabilities to commit crimes (40, 48). Vona and Kassem state that the financial pressure of individuals or organizations is the key motivation for fraud (49, 50). And the opportunity is created by ineffective control or governance systems, such as inadequate job division, weak internal control, and irregular audit (48, 51, 52). From the perspective of capability, Thompson proposed individual corruption and institutional corruption are two different phenomena in the field of healthcare (53). According to Fan, individual attribution and organizational attribution are two different interpretation paths due to insurance fraud (54). Rationalization is the attribution of improper behavior on the moral level. The existence of bad behavior or bad morals can make someone or government officials commit fraud (55). Nuswantara and Maulidi stated that a mechanism for cultural transmission of a pro-fraud attitude is influenced by the external organization (e.g., reference group), and the rate of emulation of behaviors determines the changes in fraud patterns that emerge in an organization (56).

#### Outcome variable

The loss results were recorded into two categories: “High loss” (>0.5 million) or “Low loss” (<0.5 million), according to the “Interpretation of the Supreme People Court and the Supreme People Procuratorate on Several Issues Concerning the Specific Application of Laws in Handling Criminal Cases of Fraud”, which the illegal acquisition of public and private property worth more than ¥ 0.5 million is deemed to be “extraordinarily large”.

#### Risk factors

**Figure 2:**
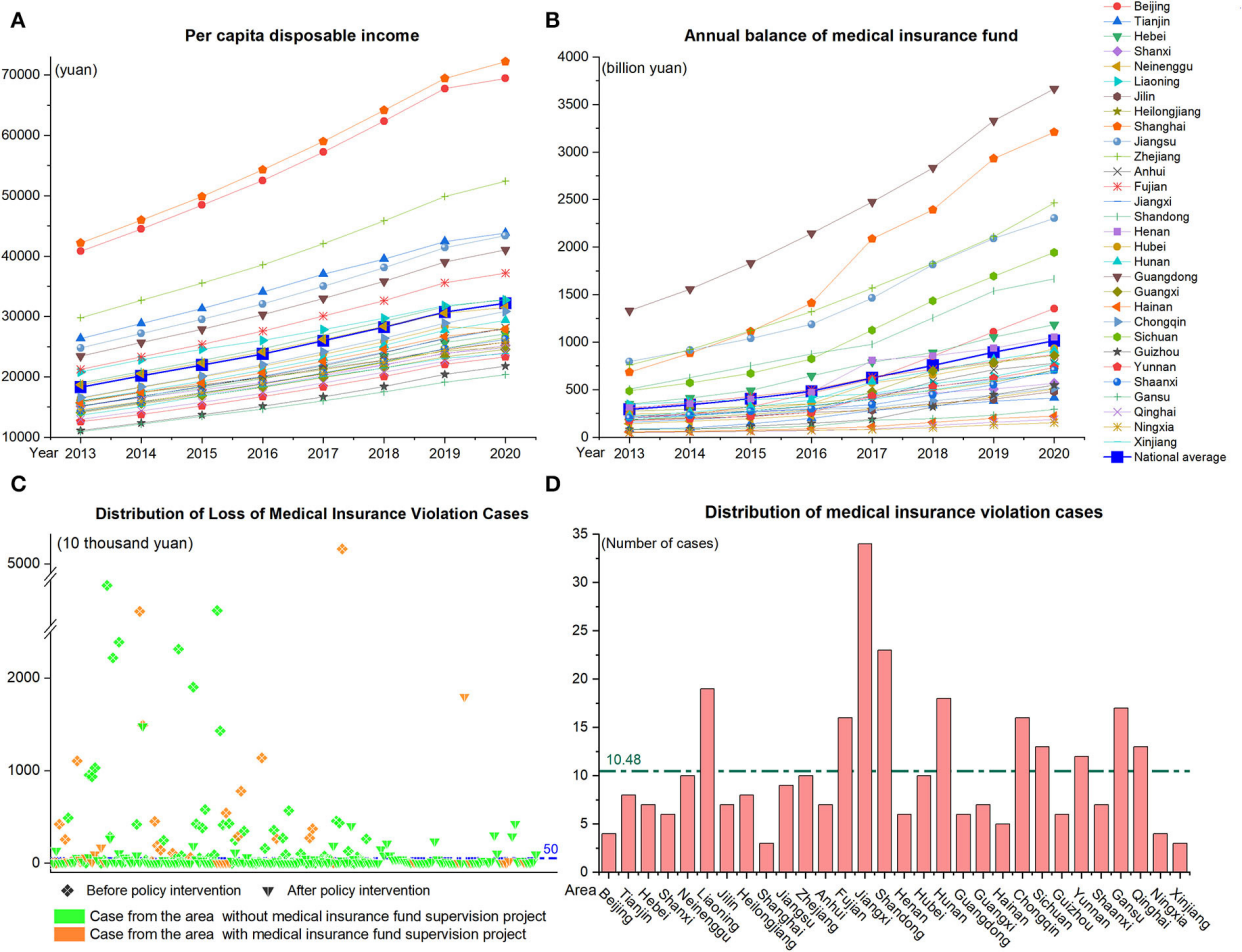
**(A–D)** Basic information of the cases.

Referring to previous studies, this study used diamond fraud theory as the analytical framework, to explore the combination of conditions leading to different levels of losses in moral hazard from the four dimensions of pressure, opportunity, capability, and rationalization. Hence, we further collected local disposable income per capita, annual medical insurance fund balances, and information supervision software projects from the National Bureau of Statistics, China Statistical Yearbook. We constructed indicators such as per capita disposable income, annual balance of medical insurance fund, informatization of supervision, implementation of regulatory policies and actions, etc., according to the four dimensions of diamond fraud theory. The definitions and details of the indicators are shown in Table 2 and Figure 2.

**Table 2:** Assignment rules for variables in cs QCA.

**Dimension**	**Variable**	**Description of attributes**	**Abbreviation**	**Value**	***N*** **^b^**
Result	Loss result	High loss: the amount involved is higher than ¥0.5 million	Result	1	82
		Low loss: the amount involved is lower than ¥0.5 million	~Result	0	188
Pressure	Per capita disposable income	Low income: PCDI in the area of cases is below average	PCDI	1	183
		High income: PCDI in the area of cases is above average	~PCDI	0	87
	Annual balance of medical insurance fund	Bad balance: BMIF is lower than the average level	ABMIF	1	178
		Good balance: BMIF is higher than the average level	~ABMIF	0	92
Opportunity	Informatization of supervision	Low degree: the area of cases did not participate in medical insurance fund supervision projects	Informatization	1	219
		High degree: the area of cases participated in medical insurance fund supervision projects	~Informatization	0	51
	Implement regulatory policies and actions	Government did not implement: before November 2018	Policy Intervention	1	108
		Government implement: after November 2018	~Policy Intervention	0	162
Capability^a^	(1) Organizational capacity	Organization	Organization	1	224
		Individual	~Organization	0	46
	(2) Categories of actors	Medical institution	Medical institution	1	167
		Non-medical institution	~Medical institution	0	103
	2) Types of medical institutions	Public medical institution	Pubmedical institution	1	90
		Private medical institution	~Pubmedical institution	0	77
Rationalization	Incident rate	High frequency: The number of local cases is above average	Incident rate	1	162
		Low frequency: The number of local cases is below average	~Incident rate	0	108

a According to the different variables of the ability dimension, the conditional combination analysis is divided into model 1 and model 2.

b Excluded cases in which the amount of loss was not reported, *N* = 270.

## Result

### Moral hazard behaviors identification in medical insurance funds

**Figure 3:**
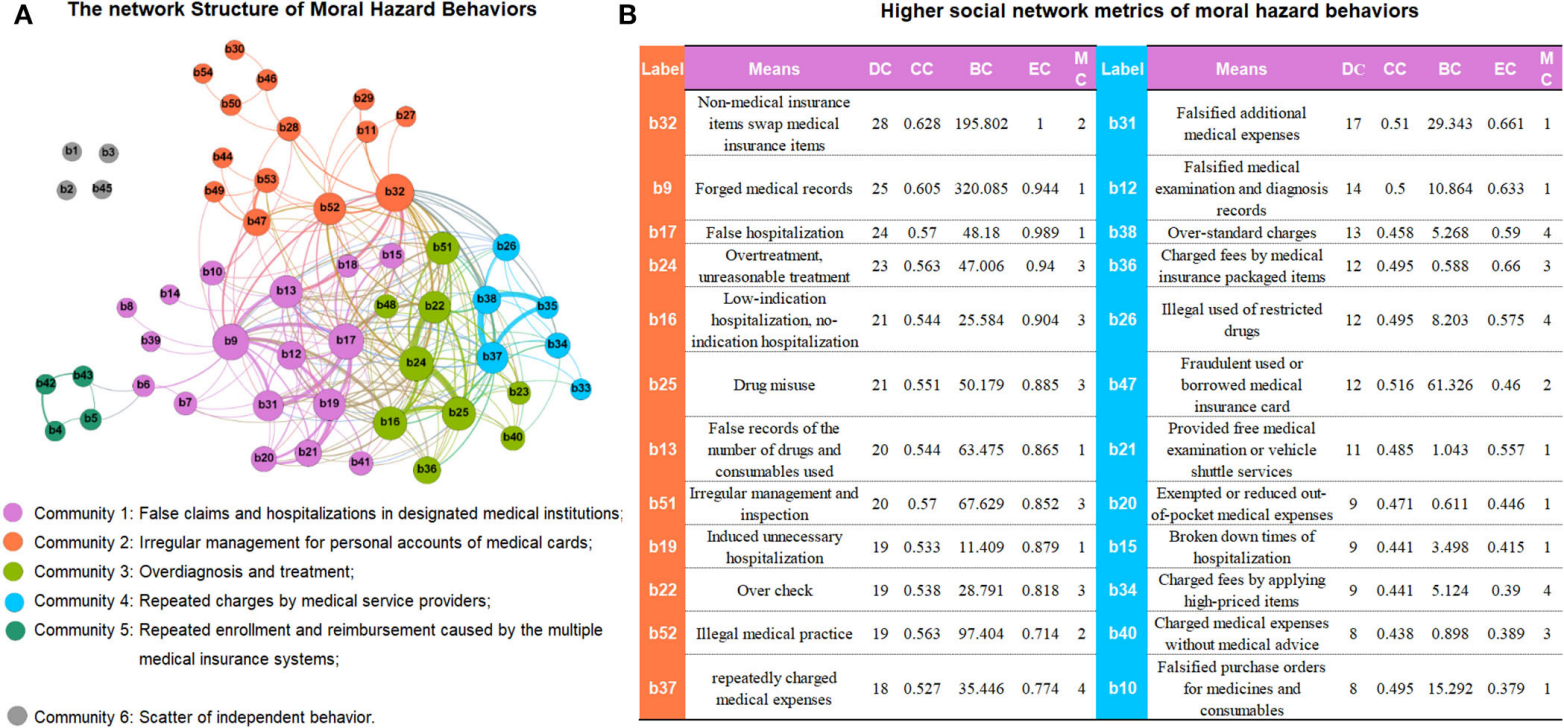
**(A,B)** The network structure of moral hazard behaviors in medical insurance fund and higher social network metrics of moral hazard behaviors. DC, degree centrality; CC, closeness centrality; BC, betweenness centrality; EC, Eigenvector Centrality; MC, modularity class. Whole detail in Supplementary File 1.

Figure 3 depicts a social network composed of 54 behavior nodes and 242 edges. The density of the network is 0.198, and the social network is relatively close, but there are still some scattered nodes (b1/b2/b3/b45), indicating that certain moral hazards are independent. Intercepts the metrics of the first 24 nodes sorted by point degree centrality of the social network, accounting for more than 80% of all nodes, which means these moral hazard behaviors are important nodes in the network. In addition, these nodes are mainly distributed in the community 1/2/3/4 (Figure 3A; Supplementary File 1).

b32 (Non-medical insurance items swap medical insurance items) is more likely to appear with other behaviors together in the same case and it links more important nodes in the network (DC_highest_ = 28, CC = 0.628, EC = 1.000). b9 (forged medical records) plays the most important intermediary role in the entire network and establishes extensive connections for other moral hazard behaviors (BC_highest_ = 320.09). Similarly, the next nodes b17 (false hospitalization), b24 (overtreatment), b16 (low-indication hospitalization), b25 (drug misuse), etc., all obtain relatively high social network parameters (Figure 3B; Supplementary File 1).

### Subjects–behaviors-problems in medical insurance moral hazard

**Figure 4:**
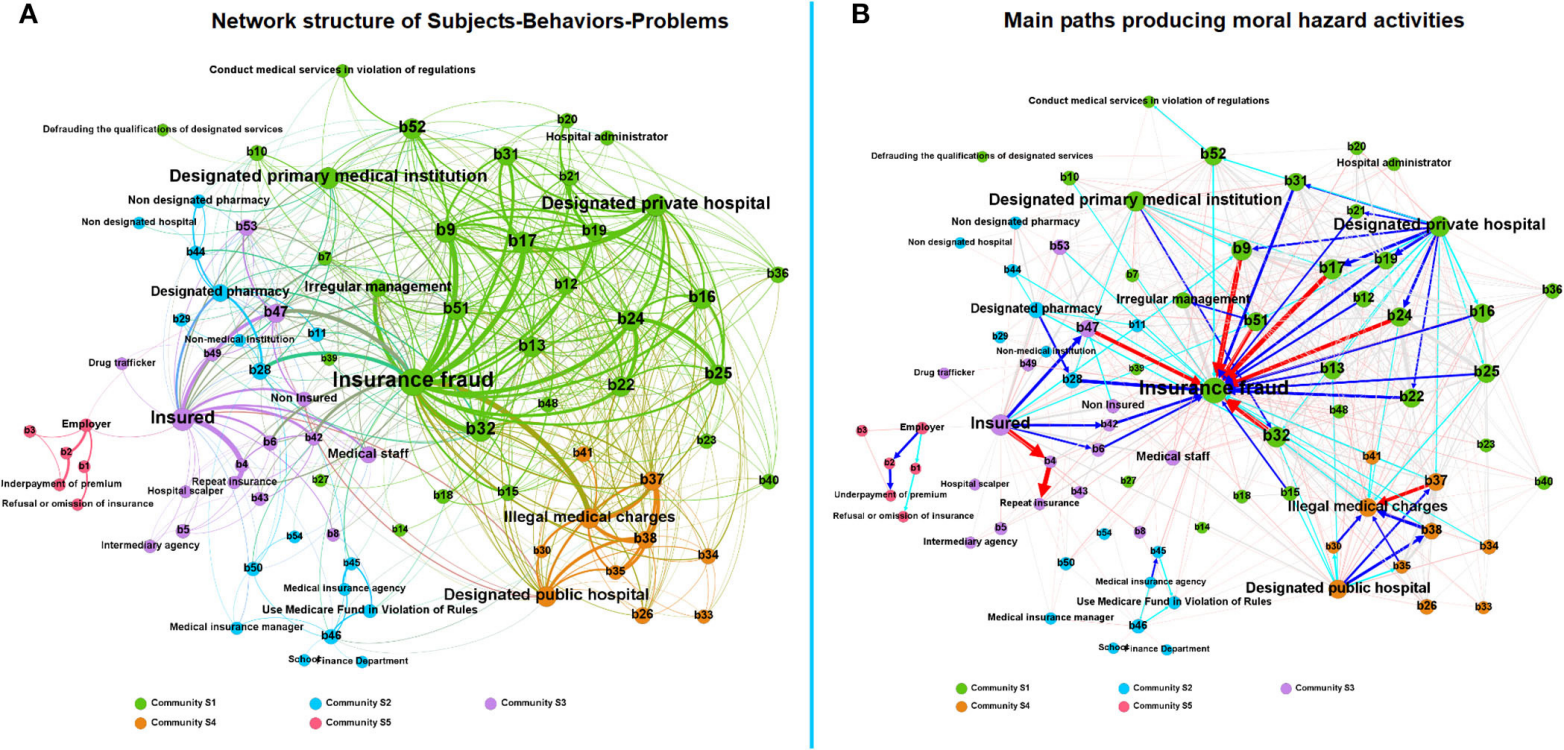
**(A)** Network structure of subjects-behaviors-problems in medical insurance moral hazard; **(B)** Main paths producing moral hazard activities. The red arrow in **(B)** means that the edge weight is ≥20, the blue arrow means that the edge weight is between 10–20, and the green arrow means that the edge weight is between 5–10.

Figure 4 and Table 3 present the network of moral hazards based on “subjects-behaviors-problems”. Designated private hospitals, designated primary medical institutions, designated public hospitals, and insured are the main institutions in the moral hazard behavior network, which produces nearly 50% of the immoral behaviors. The proportion of out-DD of designated private hospitals is 33/54 = 61%, and all have a high closeness centrality (CC > 0.8), which is the main subject of behavior diffusion (Table 3). Medical insurance fraud is the most prominent, with the highest in-DC (50) and EC (1), indicating that almost all behavior nodes are related to insurance fraud. The following problem is illegal medical charges, with in-DC (26) and EC (0.593) (Table 3; Figure 4A).

**Table 3:** Social network metrics of the subject–behavior problem of moral hazard in medical insurance (partly).

**Label**	**Out-DC**	**CC**	**MC**	**Label**	**DC**	**In-DC**	**Out-DD**	**BC**	**EC**	**MC**
Designated private hospital	33	0.851	1	b47	11	10	1	1.813	0.052	3
Designated primary medical institution	30	0.857	1	b52	11	6	5	11.344	0.031	1
Designated public hospital	27	0.865	4	b28	10	7	3	8.562	0.036	2
Insured	25	0.821	3	b22	10	7	3	2.280	0.036	1
Designated pharmacy	15	0.800	2	b9	10	6	4	2.896	0.031	1
Medical staff	13	0.760	3	b17	10	6	4	4.014	0.031	1
Hospital administrator	8	0.722	1	b51	9	6	3	1.779	0.031	1
Non Insured	5	0.692	3	b16	9	6	3	2.262	0.031	1
Non designated pharmacy	5	0.647	2	b13	9	6	3	1.779	0.031	1
Employer	4	0.700	5	b46	9	6	3	10.998	0.031	2
Medical insurance manager	3	0.615	2	b31	9	5	4	3.646	0.026	1
Intermediary agency	3	0.714	3	b25	9	5	4	2.416	0.026	1
Medical insurance agency	2	0.625	2	b32	8	5	3	0.996	0.026	1
Hospital scalper	1	0.667	3	b53	8	5	3	1.707	0.026	3
Drug trafficker	1	0.667	3	b24	8	5	3	1.266	0.026	1
Non-medical institution	1	0.571	2	b38	7	4	3	0.831	0.021	4
Non designated hospital	1	0.667	2	b12	7	3	4	1.361	0.016	1
School	1	0.571	2	b6	6	5	1	1.485	0.026	3
Finance Department	1	0.571	2	b44	6	5	1	1.341	0.026	2
**Label**	**In-DC**	**EC**	**MC**	b37	6	3	3	0.445	0.016	4
Insurance fraud	50	1.000	1	b41	6	3	3	0.611	0.016	4
Illegal medical charges	26	0.593	4	b10	5	4	1	0.172	0.021	1
Irregular management	18	0.457	1	b50	5	4	1	0.517	0.021	2
Use Medicare Fund in Violation of Rules	7	0.173	2	b15	5	3	2	0.244	0.016	1
Conduct medical services in violation of regulations	4	0.115	1	b19	5	2	3	0.366	0.010	1
Repeat insurance	3	0.041	3	b4	4	2	2	0.905	0.010	3
Underpayment of premium	2	0.019	5	b42	4	2	2	0.905	0.010	3
Refusal or omission of insurance	1	0.009	5	b45	4	2	2	1.234	0.010	2
Defrauding the qualifications of designated services	1	0.031	1	b2	2	1	1	0.500	0.005	5

Out-DC, out-degree centrality; In-DC, in-degree centrality; Whole detail in Supplementary File 2.

Figure 4B shows the main paths producing moral hazard activities by different subjects. In community S1, designated private hospitals play the main actor led in medical insurance fraud through b17 (false hospitalization), b19 (induced unnecessary hospitalization), b24 (overtreatment), and b9 (forged medical records). In addition, designated primary medical institutions commit fraud through b32 (non-medical insurance items swap medical insurance items). Community S2 consists of two scattered small groups, which performance by designated pharmacies for fraud by selling and settling daily necessities using medical insurance cards (b28), and illegal usage of medical insurance funds by medical insurance agencies through reimbursing the medical expenses beyond medical insurance payment range (b45) (Table 3; Figure 4B).

In community S3, the insured for insurance fraud by b47 (fraudulents used or borrowed medical insurance card), b6 (forged invoices), b42 (repeatedly reimbursed between different medical insurance systems), as well as b4 (repeat enrolling in health insurance). The path of Community S4 presents the unethical behavior of illegal charges in designated public hospitals by b37 (repeatedly charged medical expenses), b38 (over-standard charges), and b35 (privately set up charging items) caused by designated public hospitals (Table 3; Figure 4B).

### Core-periphery analysis of condition combinations in cs/QCA

**Figure 5:**
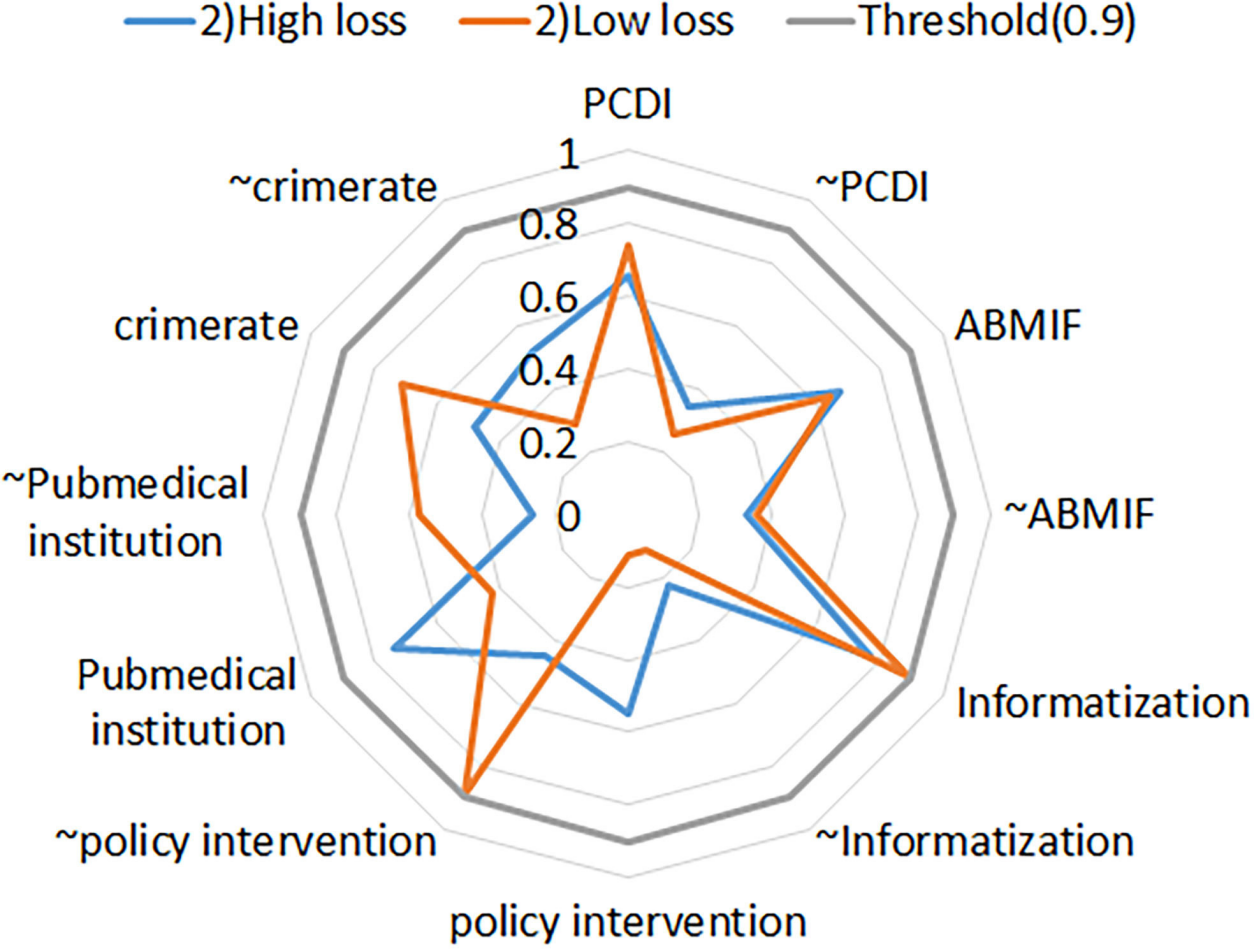
Necessity analysis of conditions in model 2.

We first conducted the sufficient necessity for conditional variable, and >0.9 is considered a necessary condition for the result (43), and the organization level meets this necessity in this study (Supplementary File 3). Combining the results of SNA mentioned above and model 1 in cs/QCA (Supplementary File 3), medical institutions are considered an important condition that caused high losses of medical insurance funds. So, we furtherly conduct model 2 to explore the condition contribution to medical insurance fund loss combination in different types of medical institutions. Figure 5 shows an analysis of sufficient and necessary conditions of the variables after the organization is removed. Figure 5 shows that the consistency scores of the variables are all <0.9, so these variables can be included in model 2 in cs/QCA (Robustness test of model 2 in Supplementary File 4).

Through the standard analysis of fsQCA software, complex solution, parsimonious solution, and intermediate solution are obtained. Model 2 excluded the necessary conditions from the condition combination analysis and constructed the truth table of remained conditions (57). By comparing the nested relationship between parsimonious and intermediate solutions, we identify the core condition of each solution: appearing in both the solutions is the core condition of the solution, and only appearing in the intermediate solution is the periphery condition (58). The consistency (High loss _consistency_ = 90; Low loss _consistency_ = 89.7) and coverage rate (High loss _coveragerate_ = 31.03; Low loss _coveragerate_ = 55.96) of the solutions at two levels of loss all have strong explanatory power and acceptability (Table 4).

**Table 4:** Core-periphery results of loss caused by medical insurance moral hazard.

**Conditions**	**High loss**				**Low loss**					
	**Hpath1a**	**Hpath1b**	**Hpath2a**	**Hpath2b**	**LpatL1**	**LpatL2**	**LpatL3**	**LpatL4**	**LpatL5**	**LpatL6**
PCDI	⨁	⨁	•	•	•	•		⨁	•	˙
ABMIF	⊕		•	•		˙	⨁		•	•
Informatization	•	•		⨁	˙		˙	•	⨁	˙
Policy intervention	•	•	•	•	⊕	⊕	⨁	⨁	⨁	⨁
Pubmedical institution	˙	˙	˙	˙	⨁	⨁		•		•
Incident rate		⨁	•		•	•	•	•	•	⨁
Raw coverage (%)	12.07	6.9	10.34	8.62	28.44	22.94	25.69	6.42	4.59	3.67
Unique coverage (%)	8.62	3.45	6.9	5.17	0	0	12.84	1.83	1.83	3.67
Consistency (%)	87.5	100	85.71	100	91.18	92.59	87.5	100	100	80
Solution coverage (%)	31.03				55.96					
Solution consistency (%)	90				89.71					

˙ Represents presence condition, ⨁ Represent absence and the blank spaces mean not necessarily present; moreover, the large • or ⨁ indicates core conditions while the small indicates peripheral conditions (57). Consistency cutoff = 0.8, frequency cutoff = 2.

Exclude cases in which violators are not medical institutions, *N* = 168 in model 2.

Four main explanation paths caused high loss of the medical insurance fund in the cases and were divided into two interpretation schemes by comparing the core conditions and the periphery conditions. One interpretation path is high-loss moral hazard behaviors caused by an enterprising opportunity type (Hpath1a, Hpath1b), which consists of high per capita disposable income (low pressure) and good health insurance fund balance (nice incentive), due to the low level of information supervision and no regulatory policy intervention (opportunities), competent actors (organizations, public hospitals) (Table 4).

Another explanation path is the risk aggressive type (Hpath2a and Hpath2b), interpreted as capable actors, who would rather take risks to obtain unreasonably high medical insurance due to economic pressure. In Hpath2a, the high incident rate in the local promotes the rationalization of moral hazard behaviors. In Hpath2b, although the informatization level of local supervision has been improved, capable public hospitals (peripheral conditions) may still operate high-loss moral hazard behaviors due to economic pressure and insufficient government supervision policies (Table 4).

Table 4 also visualizes 6 conditional combination paths caused low-loss, of 3 which explain the medical insurance moral hazard (Lpath1/2/3). Different from high-loss cases, low-loss cases mainly occur after policy interventions (core conditions). In Lpath1, low per capita disposable income is the core condition, due to local economic pressure and low level of supervision informatization (peripheral conditions), the high incident rate has become a new rationalization condition for behavior, and the private medical institution might promote the occurrence of low-loss moral hazard. In Lpath2, low-loss cases are affected by both low per capita disposable income and low medical insurance funds, regardless of the level of regulatory informatization. Similarly, in Lpath3, good medical insurance fund balances (positive incentive) and low informatization levels (periphery condition) in areas are a low-risk combination (Table 4).

### “Going back to cases”, for finding key nodes which led to high loss

“Going back to cases” is common in QCA (59). Based on different condition configurations found, we further explore the key problem of the cause by returning to cases. SNA was used to present the behavioral paths of high-loss cases, correlating the behavioral activities of the actors with the conditional configuration of the causes.

**Figure 6:**
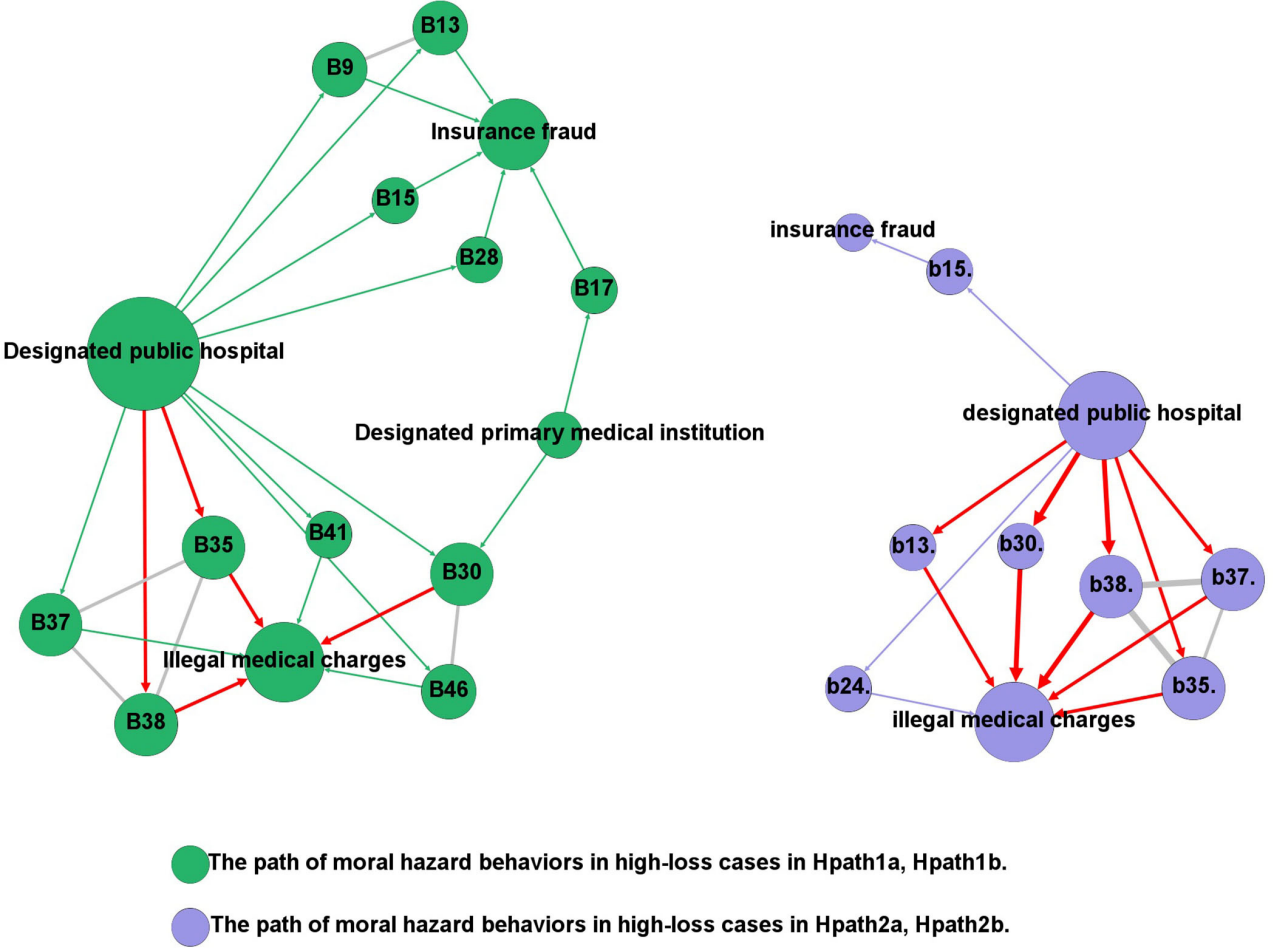
The paths of moral hazard behaviors in high-loss cases.

As shown in Figure 6, in the condition combination of Hpath1a and Hpath1b, public hospitals commit high-loss insurance fraud by forging medical records (b9), false records of drugs and consumables used (b13), decomposing hospitalization (b15), sailing and settling daily necessities through medical insurance card (b28). In addition, public hospitals also took the following actions, such as charging medical expenses repeatedly (b37), over-standard charges (b38), unreasonable charges (b41), setting up charging items privately (b35), misappropriated medical insurance funds by administrative power (b46), as well as selling medicines and consumables beyond the prescribed mark-up rate (b30). Designated primary medical institutions on the same path also caused high losses by selling medicines and medical consumables beyond the prescribed markup rate (b30), and false hospitalization (b17). So do public hospitals but additionally included overtreatment (b24) (Figure 6).

## Discussion

The purpose of the study is to explore the complex phenomena and causes of medical insurance moral hazard activities. Since the action on supervision of the medical insurance fund in November 2018, NHSA has paid more attention to the governance of medical insurance violations. This study found that the types of medical insurance moral hazard activities are diversified, and behavioral relationships are networked. In the visualization of typical cases published on official websites of the government, almost all forms of medical insurance moral hazard issues, and the association of actors can be found. It includes the basic questions and action paths of risk behaviors of various entities in the financing, management, and payment of medical insurance funds.

First, the most serious focal problem was insurance fraud. This study shows a behavioral network of health insurance fraud that medical service institutions operate illegally to obtain medical insurance funds compensation through unnecessary hospitalization, overtreatment, falsifying medical records, replacing non-medical insurance items with medical insurance items, etc. Designated private hospitals and designated primary medical institutions were the main agencies generating insurance fraud. Some studies reported on similar types of health care fraud in private hospitals in other countries and highlight the risks and management costs that insurance fraud poses to private hospitals (10, 60–62). But in China, medical insurance funds are usually managed by government departments. Designated medical institutions provide medical services to patients, and then seek financial compensation from the government. Private hospitals and primary medical institutions make significant contributions to healthcare in China. However, private hospitals follow the market economy model and adopt passive strategies in the face of moral hazards such as fraud. Compared to public hospitals, the financial difficulties facing private hospitals limit their altruistic behavior—without money, there is no mission (62). In addition, the large number of primary medical institutions in the urban-rural fringes are more like “hidden corners” of fraud that are not detected by modern monitors. Detecting fraudulent transactions in healthcare systems is a difficult task due to the complex relationships among dynamic elements including physicians, patients, and services (63). Under asymmetric medical information, a limited management team faces a large number of medical service bills, hence large-scale and effective identification of false medical behaviors and bills has become a difficult problem in China's medical insurance management.

Another problem caused by moral hazard is illegal medical charges, especially in designated public hospitals repeated medical expensing through various unreasonable items. Some scholars pointed out that moral hazards can also exist on an organizational level. Frequent triggers for moral hazards are corruption in organizations, the vulnerability of medical service supply chain management systems, and the lack of awareness of self-control fees in medical institutions (13, 64, 65). Furthermore, there are three types of problems in the financing stage, repeated insurance, refusal, or omission of insurance, as well as underpayment of premium. Repeated insurance seems to increase the medical insurance fund, but it may also cause the repeated reimbursement of medical expenses for insured patients during the payment stage. Although employers must pay medical insurance premiums for their employees by a certain percentage of their employee's wages. However, to save labor costs, employers might neglect to insure their employees. These uninsured types of employees are often marginal personnel; besides, employers will under-report the payment base (employee wages) to achieve the purpose of underpaying premiums. Those illegal operations can be attributed mainly to management loopholes and information lag. The collection of employee medical insurance premiums, funds management and medical service provision—involves multiple departments, but the information systems of each health care sector are independent. Over the past decade, a large number of healthcare moral hazard studies have been successfully conducted around the world. However, many developing countries, have not yet developed a substantive monitoring system that can quickly capture various medical moral hazard issues (63). Likewise, before launching a special campaign against fraudulent insurance, establishing a medical insurance moral hazard identification and monitoring system was not a priority for the Chinese health care sector. According to previous research, social network analysis may be a new attempt (21–24). SNA can present the complex structure and action paths of medical risk behaviors through the activity network, allowing regulators to quickly identify high-risk behaviors and key groups, to clarify the priority of governance moral hazard activities.

This research shows that the fund's loss of medical insurance moral hazard cases is related to financial pressure, opportunities, capabilities, and behavioral rationalization. High-loss cases exist in opportunism without regulatory technology, and risky adventurism with economic pressure, mainly driven by medical institutions, which mainly occurred before implemented interventional regulatory policies and campaigns were implemented by the government. Our findings are similar to those of other scholars who pointed out that regulatory policies, classification of medical institutions, income level of residents, the technical level of supervision, and the abundance of medical insurance funds are the antecedent conditions that affect moral hazard behaviors (38, 66). Opportunism seems to have always been associated with moral hazard, especially in principal-agent relationships in public service, where information asymmetry is an important advantage of organizational violations (67–69). Designated public medical institutions have a strong manipulation of medical resources and interpret medical information when providing medical services to insured persons and settling expenses with insurance institutions. As an organization with absolute control over medical resources, public hospitals might result in a high loss of moral hazard activities if the rules are not followed. As indicated by Alonazi WB, “Hospitals can minimize the moral hazards, if they wish. But if they do not wish, they can practice moral hazards secretly”. A moral hazard is not considered a criminal action so far, and the implication is an increase in the incidence of moral hazards from one organization to another (25). Thence rationalization of opportunist fraud occurs when a society loses its collective morality and other values, apart from money values, and the increase in incident rate is a sign of society's loss of collective morality (5). Risky adventurism seems to be related to the conflict between service ethics and business ethics caused by economic pressures (62). Ni Wayan Rustiarini mentioned in analyzing public procurement fraud from the perspective of diamond fraud theory, public service providers expect to be compensated through some wrongful acts when legitimate income (low income) is not commensurate with their position and responsibilities (70). In recent years, the reform of payment methods oriented to control medical costs ignored the matching of the incentive mechanism of medical service provision and the incentive mechanism of the medical insurance system; (71) And the lack of meaningful supervision laws makes the non-compliant diagnosis and treatment behavior of public hospitals in the fuzzy area of legal punishment; The opportunity for moral hazard arises when hospitals lack internal self-examination within the organization and internal control over risk management.

In addition, our study found that the government's strengthening of fund supervision is an important factor in low-loss cases. When violator's rationalization factors weaken because of policy attention strengthening, violators with different abilities will readjust their behavioral strategies according to the changing policy environment. Opportunities due to regulatory loopholes, financial pressure, organizational capacity, and new rationalization are recombined in low-loss moral hazards. The incident rate in the area may be a reasonable explanation for the unethical behavior of the actors. Shepherd and Button argue that organizational inhibitions for immoral behaviors are due to the construction of differential rationalization interpretation; wherever occupational crimes are normalized, the perception that fraud is ordinary, mundane activities, people are more likely to view them as tolerable practices (72). A study from the United States found that the prevalence of moral hazard in healthcare fraud has a contagious effect (73). As Chinese say that the law does not blame the public, which means that when a certain behavior has a certain group or universality, even if the behavior contains some illegal or unreasonable factors, the law is difficult to punish. It is worth mentioning that telecommunications technologies and the internet may contribute significantly to health care system performance (74). However, insurance companies and government agencies have yet to take full advantage of technology in regulating health insurance moral hazard because it has not yet realized its value and efficiency of it (15). Financial pressure and a low level of information technology will still make actors generate moral hazard behaviors, such as not joining the supervision of information construction projects, and still using manual auditing methods due to lacking support for big data in some economically underdeveloped areas of China.

## Limitations

There are also several limitations in our study. First, the sample size is limited. To obtain relatively standard variable information, we discarded some unqualified cases when selecting samples, so some information may be ignored. However, the selected samples are from typical cases published by the government, so they are representative to some extent. Second, in the division of the outcome variable in QCA, although it is more appropriate to calibrate continuous variables by using fs/QCA, it is difficult to find appropriate anchor points for variable calibration because of the large difference in the loss involved in cases, therefore we classified the outcome variables according to the reliable classification basis found in China's relevant laws. Finally, due to the limitation of case data, the variables used in this study may not be able to capture all the possible causal complexity of medical insurance moral hazard, and demographic characteristics such as age, gender, patient's disease category, and differences in the departments that provide inpatient services may also lead to deviations in the results (25, 75, 76). Hence, one important suggestion is that conduct research on medical insurance moral hazards of key populations.

## Conclusion

To our knowledge, this study is the first attempt to explore how moral hazard behaviors lead to the loss of medical insurance funds using a hybrid research method combining SNA and QCA. Our findings suggest that a complex and diverse network of moral hazard activity is forming around the loss of health insurance funds, which contains different actors committing various violations. Private hospitals, primary medical institutions, and public hospitals occupy the center position of the network according to several indicators of SNA and stand out among the offenders. Through these high-frequency behavior nodes such as non-medical insurance items swapping medical insurance items, forged medical records, false hospitalization, and overtreatment, these illegal medical institutions, respectively, formed two moral hazard communities with insurance fraud and illegal medical charges as core issues in the network. Through cs/QCA, we propose that both opportunism and risky adventurism would lead to high-loss cases. In the conditional configuration defined as opportunism, due to the low level of informatization and lack of policy intervention, a sufficient medical insurance fund balance will be coveted by capable medical institutions. And the conditional configuration defined as risky adventurism is associated with factors such as low levels of PCDI, low levels of ABMIF, insufficient government supervision policies, and high violation rates. This interesting finding illustrates the importance of strengthening policy intervention and informatization of supervision to reduce the loss of insurance funds. China's government is facing various challenges in governing moral hazard behaviors, and the NHSA should continue to strengthen the supervision of medical insurance funds, pay attention to areas with low-economic development and high incident rates, and focus on monitoring the behavior of major medical service providers, such as designated public hospitals, private hospitals, and designated pharmacies in the future.

## Data availability statement

The datasets used and analyzed during the current study are available from the corresponding author on reasonable request.

## Ethics statement

Ethical review and approval was not required for the study on human participants in accordance with the local legislation and institutional requirements. Written informed consent for participation was not required for this study in accordance with the national legislation and the institutional requirements.

## Author contributions

QHW was responsible for the overall design of the research and revised the paper. YHQ conducted analyze the results, and drafted the manuscript. JCL and PFG substantially contributed to data acquisition. RZW and HL assisted with the literature review. JJL and ZK contributed to the interpretation of the results and the writing of the manuscript. All authors contributed to this manuscript. All authors approved of the current version of this manuscript for publication.

## Funding

This study was supported by the National Key Social Science Fund of China (Grant No. 19AZD013).

## Conflict of interest

The authors declare that the research was conducted in the absence of any commercial or financial relationships that could be construed as a potential conflict of interest.

## Publisher's note

All claims expressed in this article are solely those of the authors and do not necessarily represent those of their affiliated organizations, or those of the publisher, the editors and the reviewers. Any product that may be evaluated in this article, or claim that may be made by its manufacturer, is not guaranteed or endorsed by the publisher.
