# Supplementary material for: How do moral hazard behaviors lead to the waste of medical insurance funds? An empirical study from China
Source: Front Public Health. 2022 Oct 26;10:988492. doi: 10.3389/fpubh.2022.988492 (PMC9643743; doi:10.3389/fpubh.2022.988492)
Supplement: Supplementary file 1 [file Data_Sheet_1.docx]

**Supplementary file 1. Social network metrics of moral hazard behaviors.**

Supplementary table 1 depicts a social network composed of 54 behavior nodes and 242 edges from Figure 2. The density of the network is 0.198, the social network is relatively close, but there are still some scattered nodes (b1/b2/b3/b45), indicating that certain moral hazards are independent; The average path length is 2.411, which means that any behavior may be related to other behaviors after an average of 2.4 nodes. The average clustering coefficient is 0.613, showing an obvious clustering effect and small group characteristics. Diversified moral hazard behaviors constitute a relatively complete and complex network. Supplementary table 2 reports the network parameters of all the behavior nodes in figure 2.

| **Supplementary table 1. Network and node-level metrics from figure 2** | |
| --- | --- |
| **Parameter** | **Value** |
| Nodes | 54 |
| Edges | 242 |
| Density | 0.198 |
| Average Degree | 9.68 |
| Average Clustering Coefficient | 0.613 |
| Resolution of Modularity | 0.274 |
| Number of Communities | 6 |
| Diameter | 7 |
| Average Path length | 2.411 |

**Supplementary table 2. Social network metrics of moral hazard behaviors from figure 2**

| **Label** | **Means** | **Frequency** | **DD** | **CC** | **BC** | **EC** | **MC** |
| --- | --- | --- | --- | --- | --- | --- | --- |
| b9 | Forged medical records | 30 | 25 | 0.605 | 320.085 | 0.944 | 1 |
| b17 | False hospitalization | 27 | 24 | 0.570 | 48.180 | 0.989 | 1 |
| b13 | False records of the number of drugs and consumables used | 15 | 20 | 0.544 | 63.475 | 0.865 | 1 |
| b19 | Induced unnecessary hospitalization | 16 | 19 | 0.533 | 11.409 | 0.879 | 1 |
| b31 | Falsified additional medical expenses | 17 | 17 | 0.510 | 29.343 | 0.661 | 1 |
| b12 | Falsified medical examination and diagnosis records | 13 | 14 | 0.500 | 10.864 | 0.633 | 1 |
| b21 | Provided free medical examination or vehicle shuttle services | 10 | 11 | 0.485 | 1.043 | 0.557 | 1 |
| b20 | Exempted or reduced out-of-pocket medical expenses | 4 | 9 | 0.471 | 0.611 | 0.446 | 1 |
| b15 | Broken down times of hospitalization | 10 | 9 | 0.441 | 3.498 | 0.415 | 1 |
| b10 | Falsified purchase orders for medicines and medical consumables | 5 | 8 | 0.495 | 15.292 | 0.379 | 1 |
| b41 | Other unreasonable charges | 7 | 7 | 0.434 | 0.160 | 0.379 | 1 |
| b7 | Fake prescription | 4 | 6 | 0.441 | 21.957 | 0.283 | 1 |
| b18 | Imposter hospitalization | 3 | 5 | 0.419 | 0.360 | 0.233 | 1 |
| b6 | Forged invoices | 12 | 4 | 0.408 | 180 | 0.081 | 1 |
| b8 | Fabricated facts about medical malpractice | 4 | 1 | 0.380 | 0 | 0.060 | 1 |
| b39 | Broken down the charge items | 1 | 1 | 0.380 | 0 | 0.060 | 1 |
| b14 | Inflated the cost of medicines and medical supplies | 1 | 1 | 0.355 | 0 | 0.054 | 1 |
| b32 | Non-medical insurance items swap medical insurance items | 29 | 28 | 0.628 | 195.802 | 1 | 2 |
| b52 | Illegal medical practice | 15 | 19 | 0.563 | 97.404 | 0.714 | 2 |
| b47 | Fraudulent used or borrowed medical insurance card | 21 | 12 | 0.516 | 61.326 | 0.460 | 2 |
| b53 | Illegal retained health insurance card | 6 | 8 | 0.445 | 21.251 | 0.306 | 2 |
| b28 | Used personal health insurance account to swipe card to sell or consume health products and daily necessities | 19 | 7 | 0.454 | 180.244 | 0.220 | 2 |
| b11 | Falsified drug sales records | 5 | 4 | 0.412 | 0.620 | 0.170 | 2 |
| b29 | Induced patients to purchase drugs | 1 | 3 | 0.405 | 0 | 0.120 | 2 |
| b49 | Reselling drugs | 4 | 3 | 0.408 | 0.984 | 0.109 | 2 |
| b46 | Misappropriated medical insurance funds with administrative power | 10 | 3 | 0.322 | 48 | 0.018 | 2 |
| b50 | Used medical insurance card to withdraw cash | 3 | 3 | 0.322 | 48 | 0.018 | 2 |
| b27 | manufactured and sold fake drugs | 1 | 2 | 0.402 | 0 | 0.109 | 2 |
| b44 | Swiped medical insurance card for non-designated medical institutions | 6 | 2 | 0.355 | 0 | 0.049 | 2 |
| b30 | Selling medicines and medical consumables beyond the prescribed mark-up rate | 12 | 1 | 0.245 | 0 | 0.002 | 2 |
| b54 | Substituted the insured person to sign the registration form | 1 | 1 | 0.245 | 0 | 0.002 | 2 |
| b24 | Overtreatment, unreasonable treatment | 23 | 23 | 0.563 | 47.006 | 0.940 | 3 |
| b16 | Low-indication hospitalization, no-indication hospitalization | 16 | 21 | 0.544 | 25.584 | 0.904 | 3 |
| b25 | Drug misuse | 15 | 21 | 0.551 | 50.179 | 0.885 | 3 |
| b51 | Irregular management and inspection | 21 | 20 | 0.570 | 67.629 | 0.852 | 3 |
| b22 | Overcheck | 17 | 19 | 0.538 | 28.791 | 0.818 | 3 |
| b36 | Charged fees by medical insurance packaged items | 3 | 12 | 0.495 | 0.588 | 0.660 | 3 |
| b40 | Charged medical expenses without medical advice | 3 | 8 | 0.438 | 0.898 | 0.389 | 3 |
| b48 | Paid kickbacks | 3 | 7 | 0.458 | 0.160 | 0.380 | 3 |
| b23 | Overdiagnosis | 3 | 6 | 0.402 | 0.143 | 0.311 | 3 |
| b37 | repeatedly charged medical expenses | 20 | 18 | 0.527 | 35.446 | 0.774 | 4 |
| b38 | Over-standard charges | 20 | 13 | 0.458 | 5.268 | 0.590 | 4 |
| b26 | Illegal used of restricted drugs | 3 | 12 | 0.495 | 8.203 | 0.575 | 4 |
| b34 | Charged fees by applying high-priced items | 5 | 9 | 0.441 | 5.124 | 0.390 | 4 |
| b35 | Privately set up charging items | 10 | 5 | 0.415 | 0.071 | 0.236 | 4 |
| b33 | Transferred the cost to medical insurance items | 2 | 3 | 0.371 | 0 | 0.129 | 4 |
| b43 | repeatedly reimbursed between different regions | 3 | 3 | 0.299 | 46.5 | 0.009 | 5 |
| b5 | Repeated participation in different coordinating areas | 2 | 3 | 0.299 | 46.5 | 0.009 | 5 |
| b42 | repeatedly reimbursed between different medical insurance systems | 15 | 2 | 0.233 | 0.5 | 0.003 | 5 |
| b4 | Repeated participation of the insured in different medical insurance systems | 28 | 2 | 0.233 | 0.5 | 0.003 | 5 |
| b1 | Employers did not apply for medical insurance for employees | 8 | 0 | 0 | 0 | 0 | 6 |
| b2 | Employers under-reported the medical insurance payment base | 13 | 0 | 0 | 0 | 0 | 6 |
| b3 | Employers Underpaid medical insurance premiums | 3 | 0 | 0 | 0 | 0 | 6 |
| b45 | Reimbursed the medical expenses beyond the scope of medical insurance payment | 11 | 0 | 0 | 0 | 0 | 6 |

**Supplementary file 2.** **Social network metrics of subject-behavior-problem of medical insurance moral hazard**

**Supplementary table 3. Social network metrics of subject-behavior-problem of medical insurance moral hazard from figure 3**

| **Label** | **Out-DC** | **CC** | **MC** |  | **Label** | **DD** | **In-DC** | **Out-DC** | **BC** | **EC** | **MC** |
| --- | --- | --- | --- | --- | --- | --- | --- | --- | --- | --- | --- |
| Designated private hospital | 33 | 0.851 | 1 |  | b47 | 11 | 10 | 1 | 1.813 | 0.052 | 3 |
| Designated primary medical institution | 30 | 0.857 | 1 |  | b52 | 11 | 6 | 5 | 11.344 | 0.031 | 1 |
| Designated public hospital | 27 | 0.865 | 4 |  | b28 | 10 | 7 | 3 | 8.562 | 0.036 | 2 |
| Insured | 25 | 0.821 | 3 |  | b22 | 10 | 7 | 3 | 2.280 | 0.036 | 1 |
| Designated pharmacy | 15 | 0.800 | 2 |  | b9 | 10 | 6 | 4 | 2.896 | 0.031 | 1 |
| Medical staff | 13 | 0.760 | 3 |  | b17 | 10 | 6 | 4 | 4.014 | 0.031 | 1 |
| Hospital administrator | 8 | 0.722 | 1 |  | b51 | 9 | 6 | 3 | 1.779 | 0.031 | 1 |
| Non Insured | 5 | 0.692 | 3 |  | b16 | 9 | 6 | 3 | 2.262 | 0.031 | 1 |
| Non designated pharmacy | 5 | 0.647 | 2 |  | b13 | 9 | 6 | 3 | 1.779 | 0.031 | 1 |
| Employer | 4 | 0.700 | 5 |  | b46 | 9 | 6 | 3 | 10.998 | 0.031 | 2 |
| Medical insurance manager | 3 | 0.615 | 2 |  | b31 | 9 | 5 | 4 | 3.646 | 0.026 | 1 |
| Intermediary agency | 3 | 0.714 | 3 |  | b25 | 9 | 5 | 4 | 2.416 | 0.026 | 1 |
| Medical insurance agency | 2 | 0.625 | 2 |  | b32 | 8 | 5 | 3 | 0.996 | 0.026 | 1 |
| Hospital scalper | 1 | 0.667 | 3 |  | b53 | 8 | 5 | 3 | 1.707 | 0.026 | 3 |
| Drug trafficker | 1 | 0.667 | 3 |  | b24 | 8 | 5 | 3 | 1.266 | 0.026 | 1 |
| Non-medical institution | 1 | 0.571 | 2 |  | b38 | 7 | 4 | 3 | 0.831 | 0.021 | 4 |
| Non designated hospital | 1 | 0.667 | 2 |  | b12 | 7 | 3 | 4 | 1.361 | 0.016 | 1 |
| School | 1 | 0.571 | 2 |  | b6 | 6 | 5 | 1 | 1.485 | 0.026 | 3 |
| Finance Department | 1 | 0.571 | 2 |  | b44 | 6 | 5 | 1 | 1.341 | 0.026 | 2 |
|  |  |  |  |  | b37 | 6 | 3 | 3 | 0.445 | 0.016 | 4 |
| **Label** | **In-DC** | **EC** | **MC** |  | b41 | 6 | 3 | 3 | 0.611 | 0.016 | 4 |
| Insurance fraud | 50 | 1.000 | 1 |  | b10 | 5 | 4 | 1 | 0.172 | 0.021 | 1 |
| Illegal medical charges | 26 | 0.593 | 4 |  | b50 | 5 | 4 | 1 | 0.517 | 0.021 | 2 |
| Irregular management | 18 | 0.457 | 1 |  | b15 | 5 | 3 | 2 | 0.244 | 0.016 | 1 |
| Use Medicare Fund in Violation of Rules | 7 | 0.173 | 2 |  | b19 | 5 | 2 | 3 | 0.366 | 0.010 | 1 |
| Conduct medical services in violation of regulations | 4 | 0.115 | 1 |  | b48 | 4 | 3 | 1 | 0.148 | 0.016 | 1 |
| Repeat insurance | 3 | 0.041 | 3 |  | b30 | 4 | 3 | 1 | 0.140 | 0.016 | 4 |
| Underpayment of premium | 2 | 0.019 | 5 |  | b23 | 4 | 3 | 1 | 0.104 | 0.016 | 1 |
| Refusal or omission of insurance | 1 | 0.009 | 5 |  | b18 | 4 | 3 | 1 | 0.104 | 0.016 | 1 |
| Defrauding the qualifications of designated services | 1 | 0.031 | 1 |  | b4 | 4 | 2 | 2 | 0.905 | 0.010 | 3 |
|  |  |  |  |  | b42 | 4 | 2 | 2 | 0.905 | 0.010 | 3 |
|  |  |  |  |  | b8 | 4 | 2 | 2 | 0.617 | 0.010 | 3 |
|  |  |  |  |  | b5 | 4 | 2 | 2 | 1.707 | 0.010 | 3 |
|  |  |  |  |  | b34 | 4 | 2 | 2 | 0.157 | 0.010 | 4 |
|  |  |  |  |  | b40 | 4 | 2 | 2 | 0.157 | 0.010 | 1 |
|  |  |  |  |  | b36 | 4 | 2 | 2 | 0.160 | 0.010 | 1 |
|  |  |  |  |  | b26 | 4 | 2 | 2 | 0.157 | 0.010 | 4 |
|  |  |  |  |  | b33 | 4 | 2 | 2 | 0.157 | 0.010 | 4 |
|  |  |  |  |  | b35 | 4 | 2 | 2 | 0.171 | 0.010 | 4 |
|  |  |  |  |  | b45 | 4 | 2 | 2 | 1.234 | 0.010 | 2 |
|  |  |  |  |  | b21 | 3 | 2 | 1 | 0.071 | 0.010 | 1 |
|  |  |  |  |  | b49 | 3 | 2 | 1 | 1.040 | 0.010 | 3 |
|  |  |  |  |  | b43 | 3 | 2 | 1 | 0.373 | 0.010 | 3 |
|  |  |  |  |  | b7 | 3 | 2 | 1 | 0.101 | 0.010 | 1 |
|  |  |  |  |  | b11 | 3 | 1 | 2 | 0.178 | 0.005 | 2 |
|  |  |  |  |  | b20 | 2 | 1 | 1 | 0.031 | 0.005 | 1 |
|  |  |  |  |  | b14 | 2 | 1 | 1 | 0.038 | 0.005 | 1 |
|  |  |  |  |  | b39 | 2 | 1 | 1 | 0.034 | 0.005 | 1 |
|  |  |  |  |  | b27 | 2 | 1 | 1 | 0.034 | 0.005 | 1 |
|  |  |  |  |  | b29 | 2 | 1 | 1 | 0.067 | 0.005 | 2 |
|  |  |  |  |  | b2 | 2 | 1 | 1 | 0.500 | 0.005 | 5 |
|  |  |  |  |  | b1 | 2 | 1 | 1 | 1.000 | 0.005 | 5 |
|  |  |  |  |  | b3 | 2 | 1 | 1 | 0.500 | 0.005 | 5 |
|  |  |  |  |  | b54 | 2 | 1 | 1 | 0.077 | 0.005 | 2 |

**Supplementary file 3. Results of cs/QCA for model 1**

As shown in Supplementary figure 1, the organization's consistency score is >0.9, so the organization is a necessary condition for the result. From the perspective of ability, compared with the individual, the ability of the organization is more likely to cause damage to medical insurance moral hazard, the consistency scores of the remaining variables are all <0.9, it is necessary to further explain the causes of the result through a combination of conditions.

**
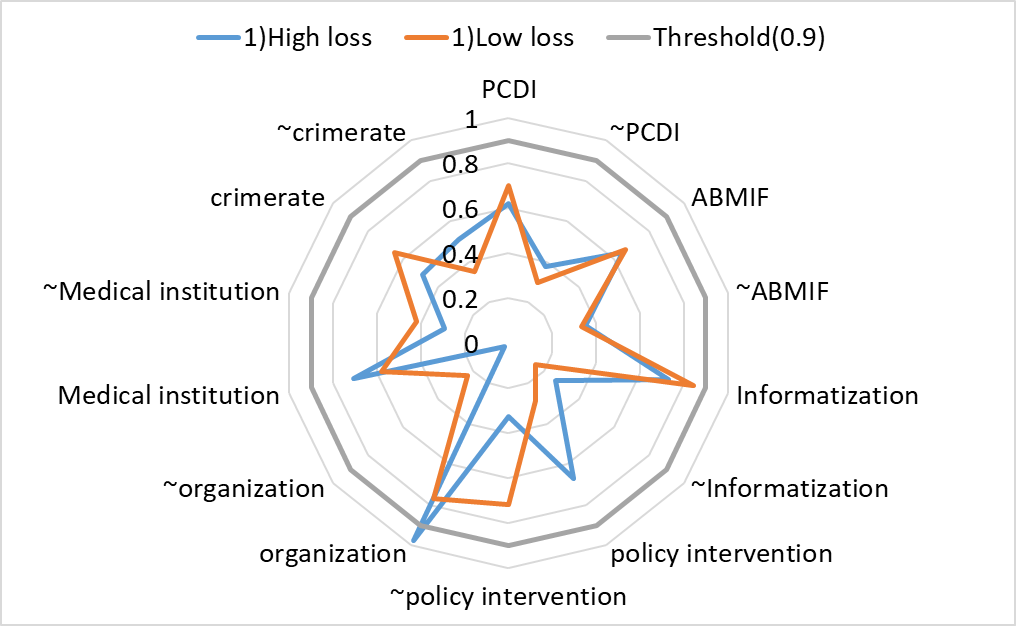
**

**Supplementary figure 1. Necessity analysis of conditions in model 1**

**Supplementary table 4. Conditional configurations for high loss in model 1**

| **Conditional configuration** | **raw  coverage (%)** | **unique  coverage (%)** | **consistency（%）** |
| --- | --- | --- | --- |
| PCDI*BMIF*policy intervention***medical institution***crime rate | 7.32 | 4.88 | 85.71 |
| ~PCDI*~BMIF*Informatization*policy intervention***medical institution***crime rate | 6.10 | 6.10 | 83.33 |
| PCDI*BMIF*~Informatization*policy intervention***medical institution** | 6.10 | 3.66 | 100 |
| ~PCDI*BMIF*Informatization*policy intervention***medical institution***~crime rate | 2.44 | 2.44 | 100 |
| ~PCDI*~BMIF*~Informatization*policy intervention***~medical institution***~crime rate | 4.88 | 4.88 | 100 |
| solution coverage (%) | 24.39 | | |
| solution consistency (%) | 90.91 | | |

Consistency cutoff = 0.8, frequency cutoff=2; N=270.

**Supplementary Table 5. Conditional configurations for low loss in model 1**

| **Conditional configuration** | **raw  coverage (%)** | **unique  coverage (%)** | **Consistency**  **（%）** |
| --- | --- | --- | --- |
| Informatization*~policy intervention*~hospitals*crime rate | 14.89 | 10.11 | 96.55 |
| PCDI*BMIF*Informatization*~hospitals*~crime rate | 7.45 | 7.45 | 82.35 |
| PCDI*~BMIF*Informatization*~hospitals*crime rate | 3.72 | 1.60 | 100 |
| PCDI*~BMIF*~Informatization*policyintervention*~hospitals*~crimerate | 1.06 | 1.06 | 100 |
| ~PCDI*~BMIF*Informatization*~hospitals*~crime rate | 2.66 | 2.66 | 100 |
| ~PCDI*BMIF*~Informatization*policyintervention*~hospitals*~crimerate | 2.13 | 2.13 | 100 |
| PCDI*BMIF*~policy intervention*hospitals*crime rate | 22.34 | 22.34 | 82.35 |
| PCDI*~BMIF*~policy intervention*hospitals*~crime rate | 3.19 | 3.19 | 85.71 |
| ~BMIF*Informatization*~policy intervention*crime rate | 19.68 | 14.89 | 90.24 |
| solution coverage (%) | 70.21 | | |
| solution consistency (%) | 88.00 | | |

Consistency cutoff = 0.8, frequency cutoff=2; N=270.

**Supplementary file 4.** **Robustness test of cs/QCA in model 2.**

To understand the stability of the solutions in qualitative comparative analysis, following the suggestions of Ragin (2008) that research can change the frequency cutoff of cases or consistency threshold for robustness test. This study conducted a robustness test, with the other processes unchanged, to change the threshold of the truth table to 1, and the consistency was 0.8. The results of solutions in the robustness test is roughly similar to the configuration in table 4 in the main text of paper, lowering the threshold increases the coverage of the solution, it is less precise than the scheme in table 4.

**Supplementary table 6. Configurations for high loss in cs/QCA results based on robustness test**

| Conditions | Hpath1 | Hpath2 | Hpath3 | Hpath4 | Hpath5 | Hpath6 |
| --- | --- | --- | --- | --- | --- | --- |
| PCDI | ⊕ | ⊕ | ● | ● | ● | ⊕ |
| ABMIF | ⊕ |  | ● | ● | ⊕ | ⊕ |
| Informatization | ● |  |  | ⊕ | ⊕ | ⊕ |
| policy intervention | ● | ● | ● |  | ● | ⊕ |
| Pubmedical institution | ● | ● | ● | ● | ⊕ | ● |
| Incident rate |  | ⊕ | ● | ⊕ | ⊕ | ● |
| raw coverage (%) | 12.07 | 10.34 | 10.34 | 6.9 | 1.72 | 1.72 |
| unique coverage (%) | 8.62 | 6.9 | 10.34 | 6.9 | 1.72 | 1.72 |
| Consistency (%) | 87.5 | 100 | 85.71 | 100 | 100 | 100 |
| solution coverage（%） | 39.66 | | | | | |
| solution consistency（%） | 92.00 | | | | | |

**● represent presence condition,⊕represent absence and the blank spaces mean not necessarily present; Consistency cutoff = 0.8, frequency cutoff=1.**

**Supplementary table 7. Configurations for low loss in cs/QCA results based on robustness test**

| Conditions | LpatL1 | LpatL2 | LpatL3 | LpatL4 | LpatL5 | LpatL6 | LpatL7 | LpatL8 | LpatL9 | LpatL10 | LpatL11 | LpatL12 |
| --- | --- | --- | --- | --- | --- | --- | --- | --- | --- | --- | --- | --- |
| PCDI | ● |  | ● | ● | ⊕ |  | ● | ⊕ | ● | ⊕ | ● | ⊕ |
| ABMIF |  | ⊕ | ● | ⊕ |  | ● | ● | ● | ⊕ | ● | ⊕ | ⊕ |
| Informatization | ● | ● |  | ● | ● | ● | ⊕ | ● | ⊕ | ⊕ |  | ● |
| policy intervention | ⊕ | ⊕ | ⊕ | ⊕ | ⊕ | ⊕ | ⊕ | ⊕ | ⊕ |  | ⊕ | ● |
| Pubmedical institution | ⊕ |  | ⊕ | ⊕ | ● | ● |  | ● |  | ⊕ | ⊕ | ⊕ |
| Incident rate | ● | ● | ● |  | ● | ⊕ | ● |  | ⊕ | ⊕ | ⊕ | ⊕ |
| raw coverage (%) | 28.44 | 25.69 | 22.94 | 9.17 | 6.42 | 4.59 | 4.59 | 2.75 | 1.83 | 1.83 | 1.83 | 0.92 |
| unique coverage (%) | 0 | 12.84 | 0 | 0 | 0 | 3.67 | 1.83 | 0 | 0.92 | 1.83 | 0 | 0.92 |
| Consistency (%) | 91.18 | 87.5 | 92.59 | 90.91 | 100 | 83.33 | 100 | 100 | 100 | 100 | 100 | 100 |
| solution coverage（%） | 62.39 | | | | | | | | | | | |
| solution consistency（%） | 90.67 | | | | | | | | | | | |

**● represent presence condition,⊕represent absence and the blank spaces mean not necessarily present;** **Consistency cutoff = 0.8, frequency cutoff=1.**
